# Differential miRNA expression profiling reveals miR-205-3p to be a potential radiosensitizer for low- dose ionizing radiation in DLD-1 cells

**DOI:** 10.18632/oncotarget.25405

**Published:** 2018-05-29

**Authors:** Rodrigo Andaur, Julio C. Tapia, José Moreno, Leopoldo Soto, Ricardo Armisen, Katherine Marcelain

**Affiliations:** ^1^ Departamento de Oncología Básico-Clínica, Facultad de Medicina, Universidad de Chile, Santiago, Chile; ^2^ Comisión Chilena de Energía Nuclear, Santiago, Chile; ^3^ Center for Research and Applications in Plasma Physics and Pulsed Power, P4, Talca, Chile; ^4^ Departamento de Ciencias Físicas, Universidad Andres Bello, Santiago, Chile; ^5^ Centro de Investigación y Tratamiento del Cáncer, Facultad de Medicina, Universidad de Chile, Santiago, Chile; ^6^ Current Address: Center of Excellence in Precision Medicine, Pfizer, Santiago, Chile

**Keywords:** radiosensitivity, low-dose ionizing radiation, miRNA, miR-205-3p

## Abstract

Enhanced radiosensitivity at low doses of ionizing radiation (IR) (0.2 to 0.6 Gy) has been reported in several cell lines. This phenomenon, known as low doses hyper-radiosensitivity (LDHRS), appears as an opportunity to decrease toxicity of radiotherapy and to enhance the effects of chemotherapy. However, the effect of low single doses IR on cell death is subtle and the mechanism underlying LDHRS has not been clearly explained, limiting the utility of LDHRS for clinical applications. To understand the mechanisms responsible for cell death induced by low-dose IR, LDHRS was evaluated in DLD-1 human colorectal cancer cells and the expression of 80 microRNAs (miRNAs) was assessed by qPCR array. Our results show that DLD-1 cells display an early DNA damage response and apoptotic cell death when exposed to 0.6 Gy. miRNA expression profiling identified 3 over-expressed (miR-205-3p, miR-1 and miR-133b) and 2 down-regulated miRNAs (miR-122-5p, and miR-134-5p) upon exposure to 0.6 Gy. This miRNA profile differed from the one in cells exposed to high-dose IR (12 Gy), supporting a distinct low-dose radiation-induced cell death mechanism. Expression of a mimetic miR-205-3p, the most overexpressed miRNA in cells exposed to 0.6 Gy, induced apoptotic cell death and, more importantly, increased LDHRS in DLD-1 cells. Thus, we propose miR-205-3p as a potential radiosensitizer to low-dose IR.

## INTRODUCTION

Radiotherapy (RT) is the standard treatment for most cancers, including colorectal cancer (CRC). It has been estimated that 60% of patients with solid cancers have received or will receive RT at least once at some point during their disease, and for 15% of these patients RT is the only treatment that will receive [1, 2]. Conventional RT protocols for a localized solid tumor include the administration of high-dose (60–70 Gy) ionizing radiation (IR), delivered in about 30 to 35 doses (2 Gy per day). These protocols are very effective, but are not free of toxicity and secondary side effects [3, 4–7].

The linear-quadratric (LQ) model has been widely used to predict the effects on cell survival after the exposure to IR. This model is generally used for calculating the effect of doses in a RT treatment. The curvilinear approach of LQ model show a correlation between cell death and IR at doses ≥ 2 Gy [1–4], assuming little or even no effect at lower doses (≤ 1 Gy) [8–11]. However, an increase in cell death has been observed in a tight range of low doses (0.2 to 0.6 Gy [12, 13]). This phenomenon, known as low doses hyper-radiosensitivity (LDHRS), is followed by an increase in radioresistance at dose closer to 1 Gy [9, 14–17]. LDHRS has been observed in ∼75% of the 50 cell lines tested *in vitro* to date including, for example colorectal (HT29 and RKO) [18, 19], bladder (RT112) [20], lung (A549) [21], melanoma (MeWo) [22] among others. In addition, LDHRS has been also shown in Multicellular tumor spheroids (MCTSs) built up with breast cancer cells [17] and also in non-tumor cells such as fibroblast, keratinocytes and lung epithelial cells [23]. This LDHRS phenomenon appears as an opportunity to decrease the IR doses used in RT [9, 11, 15, 24–26], decreasing toxicity and side effects of conventional therapy. In addition, it was reported that serum from 0.3–0.03 Gy irradiated DBA/2 mice allowed an increased radioresistance and viability of non-irradiated breast and glioblastoma cell lines [27], which suggested that exposure to low doses IR would also diminish bystander effect of RT.

Even though LDHRS is very efficient in killing cells per dose unit, [1, 21, 25, 28] the total cytotoxic effects gained with such low doses are not enough to achieve therapeutic effect in a single low-dose fraction. However, its benefit has been successfully exploited by using Low Doses Fractionated Radiotherapy (LDFRT). In this sense, spreading the total dose into short, low-dose pulses has been shown to effectively limit the undesirable tissue toxicity as well as to reduce complications [29–31].

Nevertheless, when radiation is used alone as LDFRT, complications are minimized, but the final clinical outcome is not necessarily improved. Importantly, preclinical as well as clinical studies have reported that using LDFRT in a chemo-radiotherapy regimen enhances the effect of chemotherapy, achieving maximum tumor cell killing with significantly reduced toxicity [1, 31–33].

Thus, pulsed low dose fractionated radiation has been validated in pre-clinical and clinical studies, although the molecular basis of reduced necrosis and preserved normal tissue integrity has remained unclear [29]. Given that low-dose IR causes DNA damage [34], LDHRS has been associated with a DNA damage response. However, it has been reported that damaged DNA in fibroblasts is repaired before 24 hours [35], thus the exact mechanism inducing LDHRS remains unknown. Understanding the molecular mechanism behind LDHRS would give an opportunity to potentiate its beneficial effects either standing alone or in radio-chemotherapy regimens. This could be achieved through biological strategies to further enhance the effectiveness and efficiency of RT or by identifying tumor biomarkers that could allow a more precise selection of the better regime for each individual patient [36].

Considering the complexity of the cellular response to IR, it is reasonable to hypothesize that one type of molecules that could be involved in the mechanism of LDRHS were microRNAs (miRNAs or miRs), given their broad effect on gene expression. These are a class of non-coding, endogenous, short (∼22 nucleotides) and single-stranded RNAs that act at the post-transcriptional level as regulators of gene expression. They bind to the untranslated region of mRNA targets, inducing either their degradation or translational repression [37, 38]. Because of its role in the regulation of gene expression, miRNAs play a key role in different cellular processes. Several studies have evaluated the impact of high-dose IR on miRNA expression, with little attention paid to the effects of low doses. For instance, it has been reported that human colonic epithelial cells modulate miRNA expression in response to high-dose IR (> 2 Gy) [39]. In addition, transfection with mimetic miRNAs, such as miR-31-5p [40], miR-100 [41], miR-630 [42] and miR-124 [43], or inhibition of miR-622 [44] and miR-221 [45], resulted in an increase of radiosensitivity at high- dose IR (4 Gy) in several CRC cell lines.

Changes in miRNA profiles after exposure to low-dose radiation have also been reported [46–50]. However, modulation of miRNA expression and its effects on radiosensitivity in a LDRHS context has not been completely explored. In this study, we evaluated LDHRS and analyzed the expression of a panel of 80 miRNAs, all related to cell proliferation or cell death, in DLD-1 human colorectal cancer cells exposed to 0.6 and 12 Gy. Our results show that five miRNAs (3 up-regulated and 2 down-regulated) are differentially expressed in low-dose irradiated DLD-1 cells. Moreover, overexpression of one of them, miR-205-3p, induced cell death and increased radiosensitivity to low-doses in DLD-1 cells.

## RESULTS

### DLD-1 colorectal cancer cells show LDHRS at 0.6 Gy

**Figure 1:**
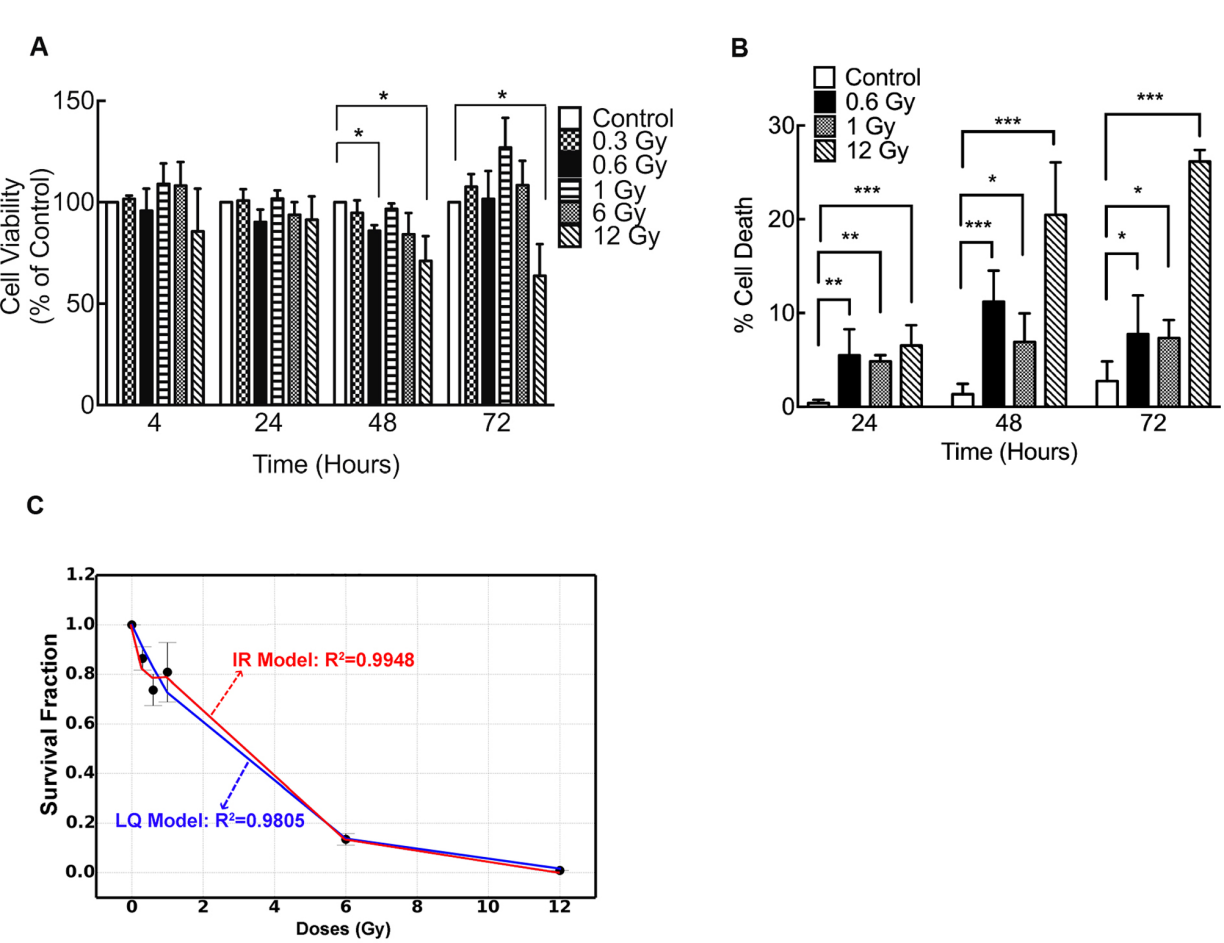
Low doses of IR reduce viability of DLD-1 cells (**A**) Viability of DLD-1 cells determined by MTS assay at different times and different doses of irradiation. Results are expressed as a percentage of control at each time point. (**B**) Cell death was evaluated with trypan blue dye exclusion assay. Data from clonogenic assay was modeled using (**C**) lineal-quadratic (LQ) model and induced-repair (IR) model. In A and B, results are expressed relative to control. Means ± S.D. of at least 3 independent experiments are shown. ^*^P < 0.05; ^**^P < 0.01; ^***^P < 0.001, two-way ANOVA.

To assess whether DLD-1 colorectal cancer cells displayed LDHRS, first they were irradiated with low (0.3 and 0.6 Gy) or high radiation doses (1, 6 and 12 Gy) and viability was evaluated by MTS at 24, 48 and 72 h after IR. At 48 h post IR, a significant reduction in viability was detected in cells exposed to 0.6 Gy and 12 Gy compared to control cells (0 Gy) (Figure 1A). As comparison and as previously reported, HT-29 cells were irradiated using the same doses, which showed a slight decrease in viability (∼10%) at 0.3–0.6 Gy (Supplementary Figure 2) [15, 18, 51].

A decrease in DLD-1 viability upon IR was confirmed by trypan blue exclusion assay. In cells exposed to 0.6 Gy the most significant increase in cell death was observed 48 h after IR, supporting previous results (Figure 1B). At this time point, cells exposed to 0.6 Gy displayed higher sensitivity than cells exposed to 1 Gy (Figure 1B). To confirm radiosensitivity in DLD-1 cells, a clonogenic assay was performed. As expected, high doses of IR elicited an important cell death but, more importantly, cells exhibited a decrease in survival at 0.3 and 0.6 Gy along with an increase in radioresistence at 1 Gy (Figure 1C), suggesting that LDHRS occurred [9, 13, 14]. In order to confirm LDHRS in these cells, data from clonogenic assay was mathematically modeled using the linear-quadratic [8, 52] and induced-repair models [10, 21, 31] (Figure 1C). Determination coefficients, R^2^, showed a better fitted curve when the induced-repair model was used. Furthermore, initial slope derived from induced-repair model (*a*_*s*_) was greater than initial slope value (*a*_*r*_) extrapolated from linear-quadratic model (*a*_*s*_/*a*_*r*_ = 3.4). All this data confirm the occurrence of LDHRS in these cells.

**Figure 2:**
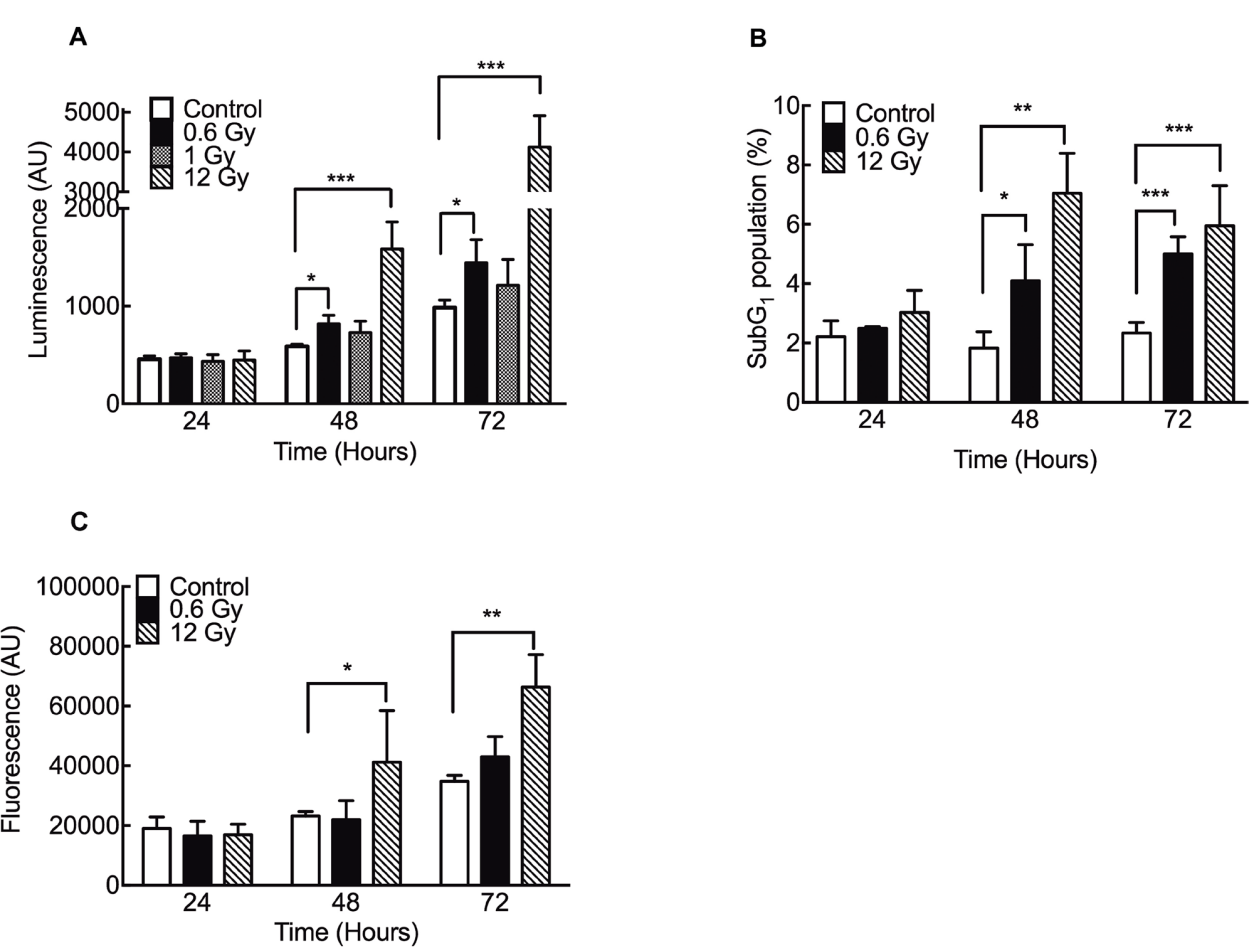
Low doses of IR increase apoptosis in DLD-1 cells (**A**) Caspase 3/7 activity was evaluated using a luminescence assay. (**B**) SubG_1_ population was evaluated by flow cytometry. (**C**) Citotoxicity was evaluated by release of intracellular proteases using a fluorometric kit detection. Results are expressed relative to control. Means ± S.D. of at least 3 independent experiments are shown. ^*^P < 0.05; ^**^P < 0.01; ^***^P < 0.001, two-way ANOVA.

To assess whether low-dose IR-induced cell death was a result of apoptosis, we evaluated caspase 3/7 activation in IR cells. Our results showed that 0.6 Gy induced a significant increase in caspase 3/7 activity at 48 and 72 h after IR (Figure 2A). Likewise, an increase in DNA fragmentation was also evidenced by an enrichment of the SubG_1_ cell population (Figure 2B). Importantly, no cytotoxicity (evaluated by protease release) was found in cells exposed to 0.6 Gy (Figure 2C). Unlike 0.6 Gy, higher doses (12 Gy) triggered both apoptotic and necrotic cell death (Figure 2).

### An early DNA damage response is elicited at 0.6 Gy

**Figure 3:**
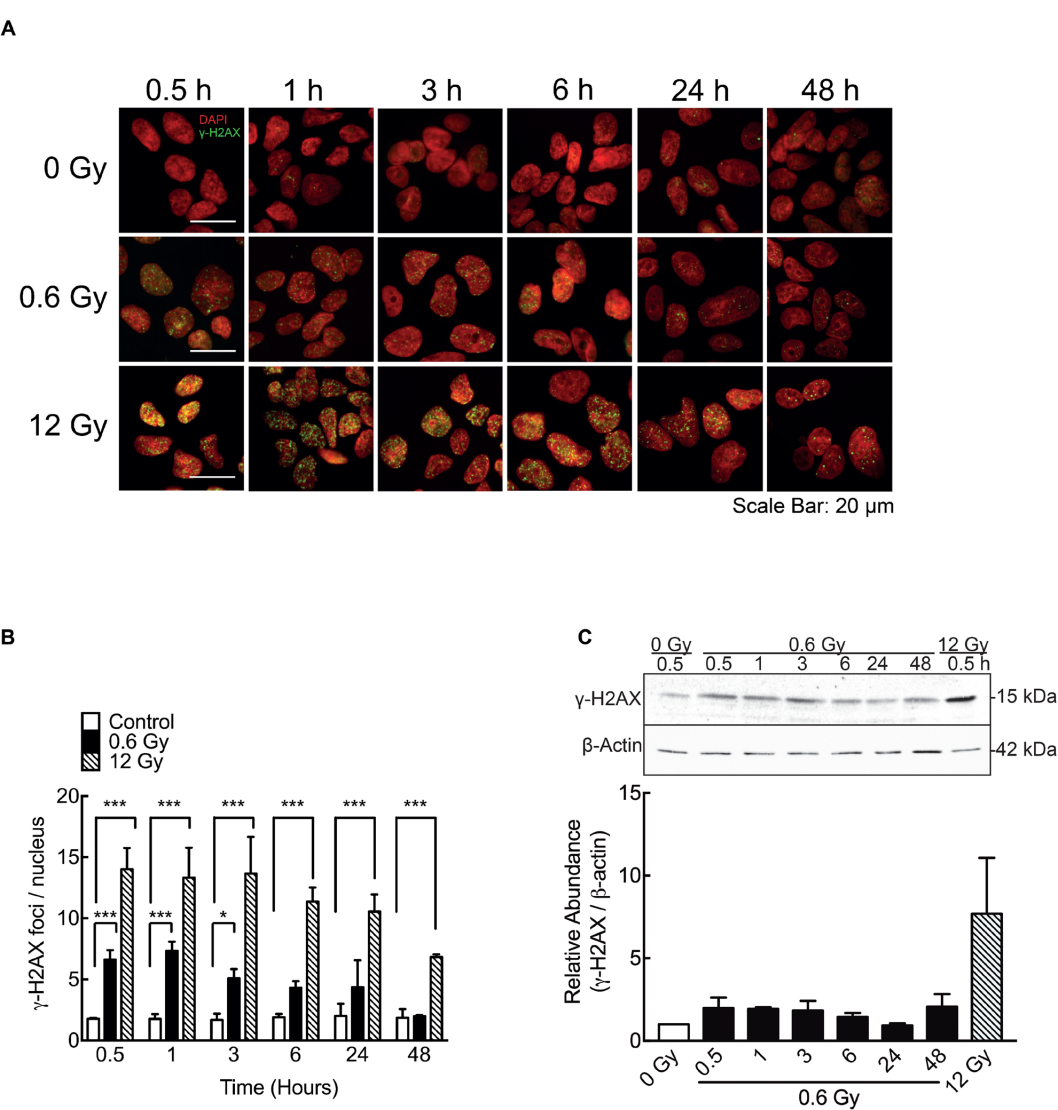
IR-induced DSB in DLD-1 cells (**A**) Evaluation of double strand break (DSBs) by immunofluorescence staining for γ-H2AX 48 h after irradiation. Nuclei were stained with DAPI. (**B**) γ-H2AX foci (Green) were quantified and normalized to the number of nuclei (Red). Cells were counted in 5 different fields and at least 100 cells were evaluated per sample. (**C**) A representative blot of γ-H2AX expression is shown along with a graph of the densitometry assay of the signal. The γ-H2AX signal was normalized to β-actin (loading control). Data represent the means ± S.D. of at least 3 independent experiments. ^*^P < 0.05, ^***^P < 0.001, Two-way ANOVA.

**Figure 4:**
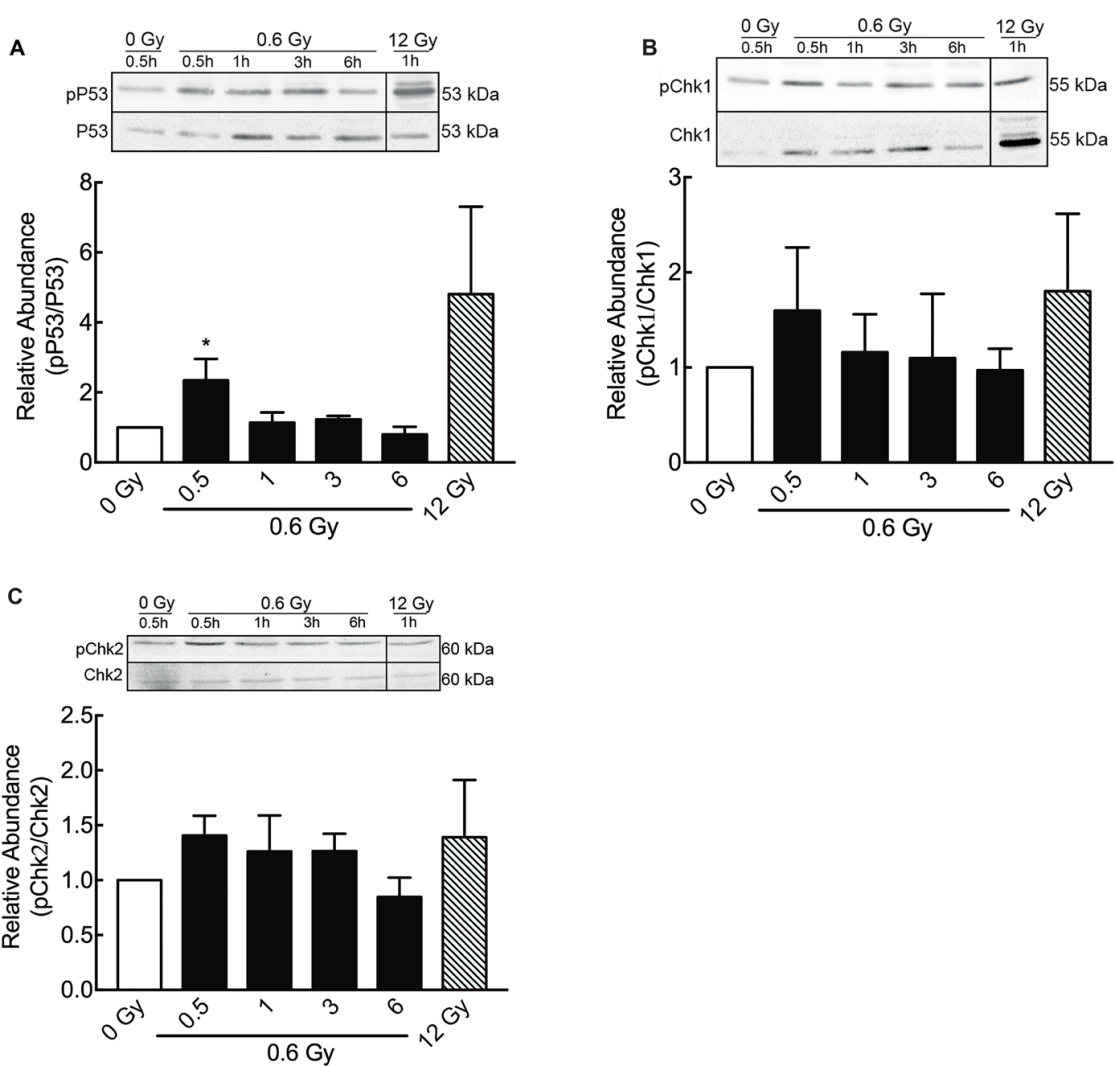
Low doses of radiation activate DNA damage repair proteins (**A**) Phosphorylation of P53 (ser 15, Figure 3, A), pCHK1 (**B**) and pCHK2 (**C**) at different times after 0.6 Gy of radiation, was evaluated by western blot. Signals of pP53, pChk1 and pChk2 were normalized by their respective total proteins. Data represent means ± S.D. of at least 3 independent experiments. ^*^P < 0.05, ^***^P < 0.001, Two-way ANOVA shown as fold changes relative to control.

In order to confirm previous data showing DNA damage response in other cell models exposed to low doses IR [53, 54] phosphorylation of H2AX histone (γ-H2AX) -a DSB surrogate marker- was analyzed. In cells exposed to 0.6 Gy, γ-H2AX nuclear foci were detected 30 min after IR and the number of foci becomes to decrease 3 h after IR (Figure 3A and 3B). Thus, 6 h after IR, no significant differences between irradiated and control cells (0 Gy) were observed (Figure 3A and 3B). These results were confirmed by a western blot assay of γ-H2AX at same times (Figure 3C). On the other hand, in cells exposed to 12 Gy, elevated γ-H2AX levels and nuclear foci were still persistent 48 h after IR (Figure 3). Along with γ-H2AX foci formation, early but slight increase in Ser15-phosphorylation of P53 (Figure 4A), phospho-CHK1 (Figure 4B) and phospho-CHK2 were also observed (Figure 4C), indicating the activation of a DNA damage response.

### High and low IR doses induce differential microRNA expression

**Figure 5:**
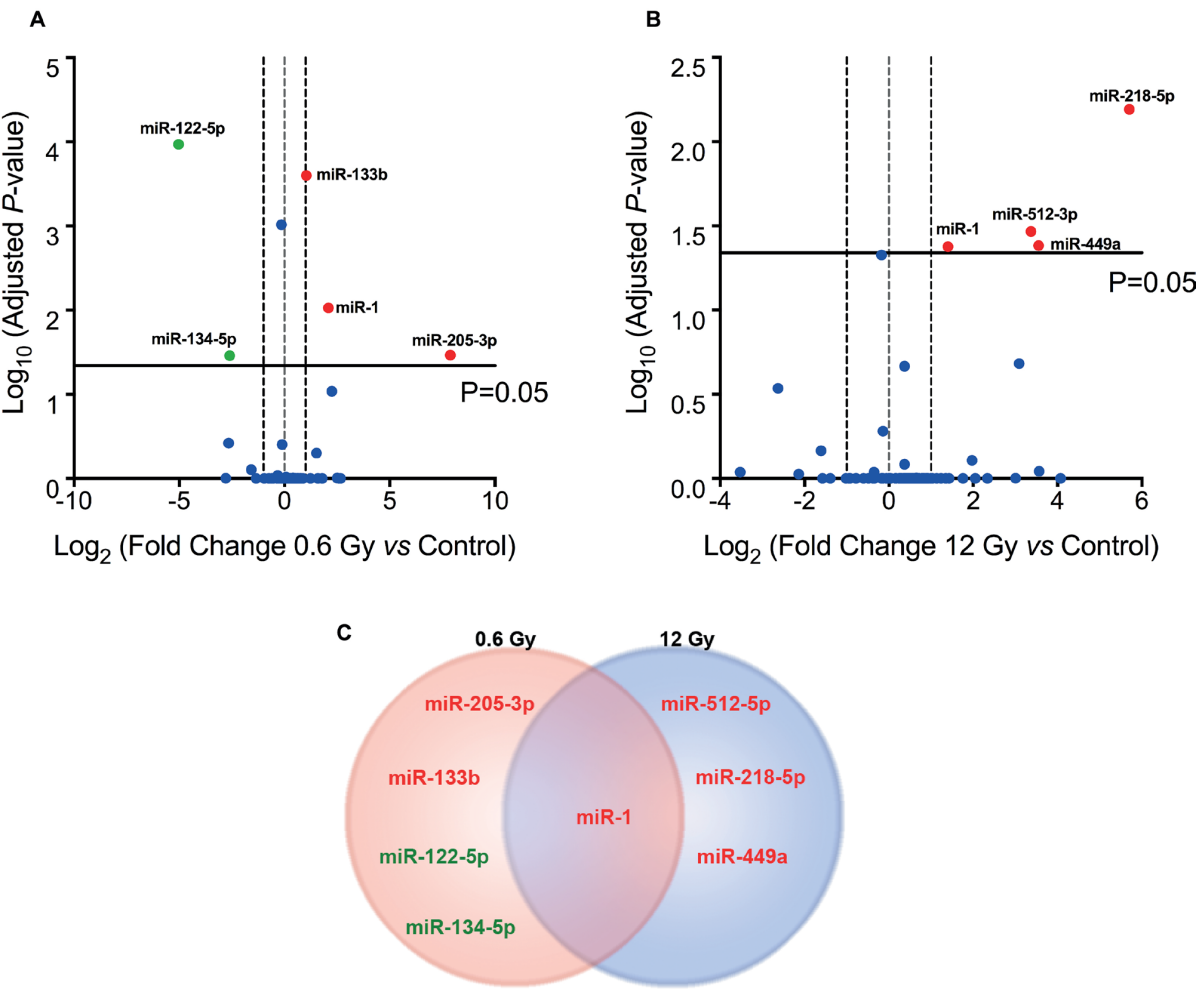
IR-induced miRNA expression profile in colorectal cancer cells Volcanoplot of miRNA expression profile obtained from a PCR array of DLD-1 cells, 48 h after exposure to (**A**) 0.6 Gy and (**B**) 12 Gy compared to non-irradiated cells (control). Overexpressed miRNAs (Fold Change ≥2×, red spot) and underexpressed (Fold change ≤2, green spot) with adjusted *P* < 0.05 are labeled. (**C**) Venn diagram comparing differentially expressed miRNAs in DLD-1 cells irradiated with 0.6 and 12 Gy.

In order to study whether miRNAs could be involved in the mechanism of LDHRS, cells were IR with 0.6 and 12 Gy and, 48 h later, qPCR array was performed to evaluate the expression of a subset of 86 miRNAs (80 associated with proliferation and apoptosis; and 6 housekeeping genes) (Supplementary Table 1). When compared to the control group, five differentially expressed miRNAs (adjusted *P* < 0.05) were identified in cells IR with 0.6 Gy, of which three were augmented (miR-205-3p, miR-1 and miR-133b) and two diminished (miR-122-5p and miR-134-5p) (Figure 5A and Table 1). On the other hand, only 4 miRNAs were differentially incremented (miR-512-5p, miR-218-5p, miR-449a and miR-1) with no differentially decreased miRNAs in cells exposed to 12 Gy (Figure 5B and Table 2). Interestingly, only one miRNA (miR-1) was deregulated to both radiation doses (0.6 and 12 Gy), indicating that most of miRNAs levels studied are IR dose-dependent (Figure 5C).

**Table 1:** Fold change and adjusted *p* value of microRNAs differentially expressed at 0.6 Gy

miRNAs Over-expressed		
miRNA	Fold Change	Adj *P*-value
miR-205-3p	225.972	0.034128
miR-1	4.084	0.009389
miR-133b	2.05	0.000251

miRNAs Under-expressed		
miRNA	Fold Change	Adj *P*-value
miR-134-5p	–6.3203	0.034752
miR-122-5p	–34.0598	0.000107

**Table 2:** Fold change and adjusted *p* value of microRNAs differentially expressed at 12 Gy

miRNAs Over-expressed		
miRNA	Fold Regulation	Adj *P*-value
miR-218-5p	5.7	0.00643
miR-449a	3.55	0.04143
miR-512-5p	3.37	0.03414
miR-1	1.4	0.04202

**Figure 6:**
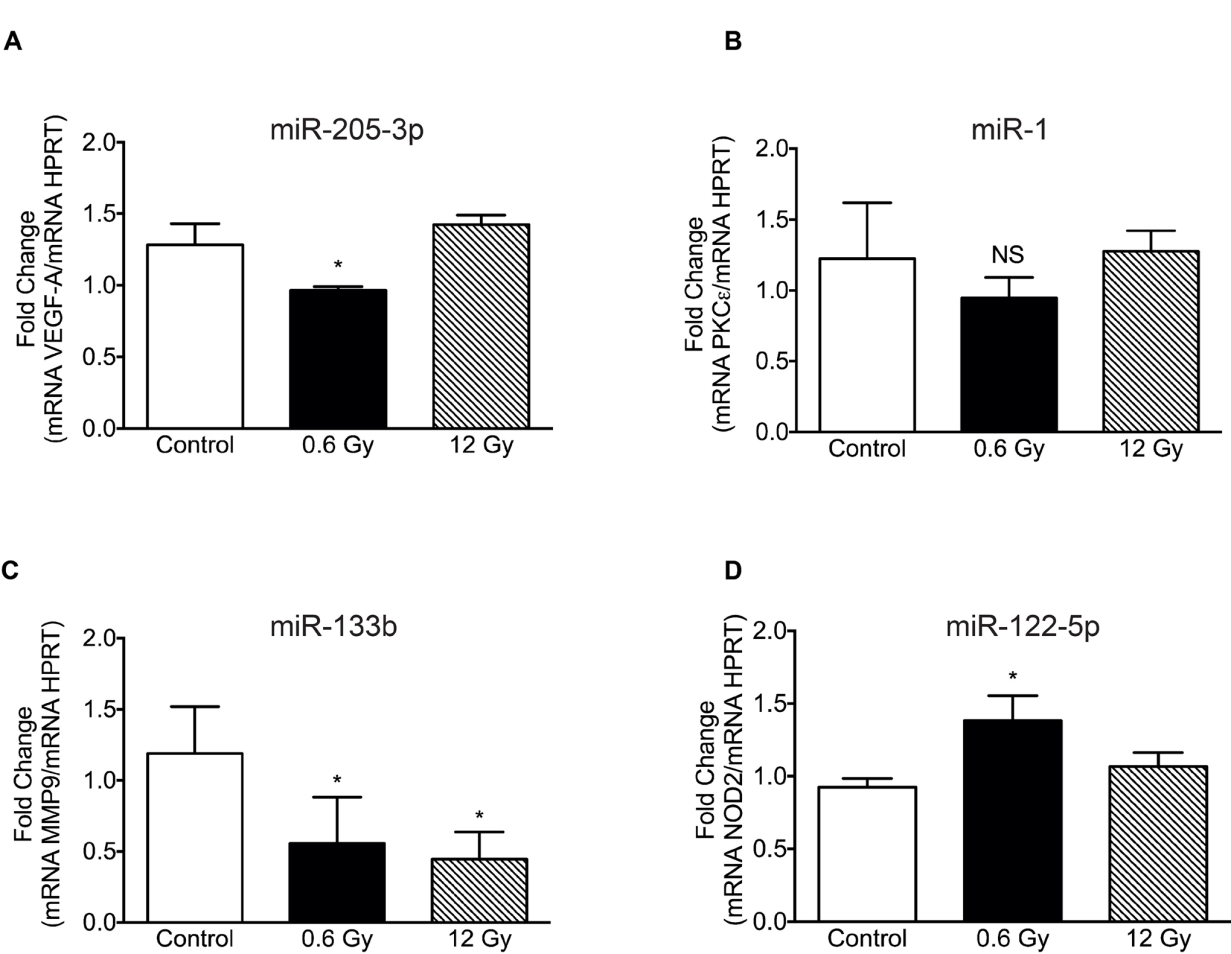
Expression levels of target mRNA in cells exposed to low and high-dose IR mRNAs targeted by: (**A**) MiR-205-3p (VEGFA), (**B**) miR-1 (PKCε), (**C**) miR-133b (MMP9) and (**D**) miR-1225p (NOD2), were evaluated by RTqPCR in DLD-1 cells, 48 h after irradiation with 0 (Control), 0.6 and 12 Gy. Expression levels were normalized to HPRT housekeeping gene. Means ± S.D of at least 3 independent experiments are shown. Results are presented relative to control. ^*^P < 0.05, Two-way ANOVA.

In order to validate our findings, RT-qPCR were carried out to assess the level of mRNA targets of the most differentially regulated miRNAs: VEGF-A for miR-205-3p (predicted with miRbase and TargetScan 7.1) (Figure 6A), PKCε for miR-1 [55] (Figure 6B), MMP9 for miR-133b [56] (Figure 6C) and NOD2 [57] (Figure 6D) for miR-122-5p. As expected, targets of the most augmented miRNAs at 0.6 Gy showed reduced mRNA levels (Figure 6A–6C), while the miR-122-5p target, NOD2, showed an increased mRNA level (Figure 6D). On the contrary, high doses of 12 Gy IR only showed impact on MMP9 mRNA levels (Figure 6C), with no effect on the expression of the other targets analyzed.

Altogether, our data shows that DLD-1 cells display LDHRS, which is associated with differential miRNA and targets profiles.

### MiR-205-3p overexpression increases LDHRS.

**Figure 7:**
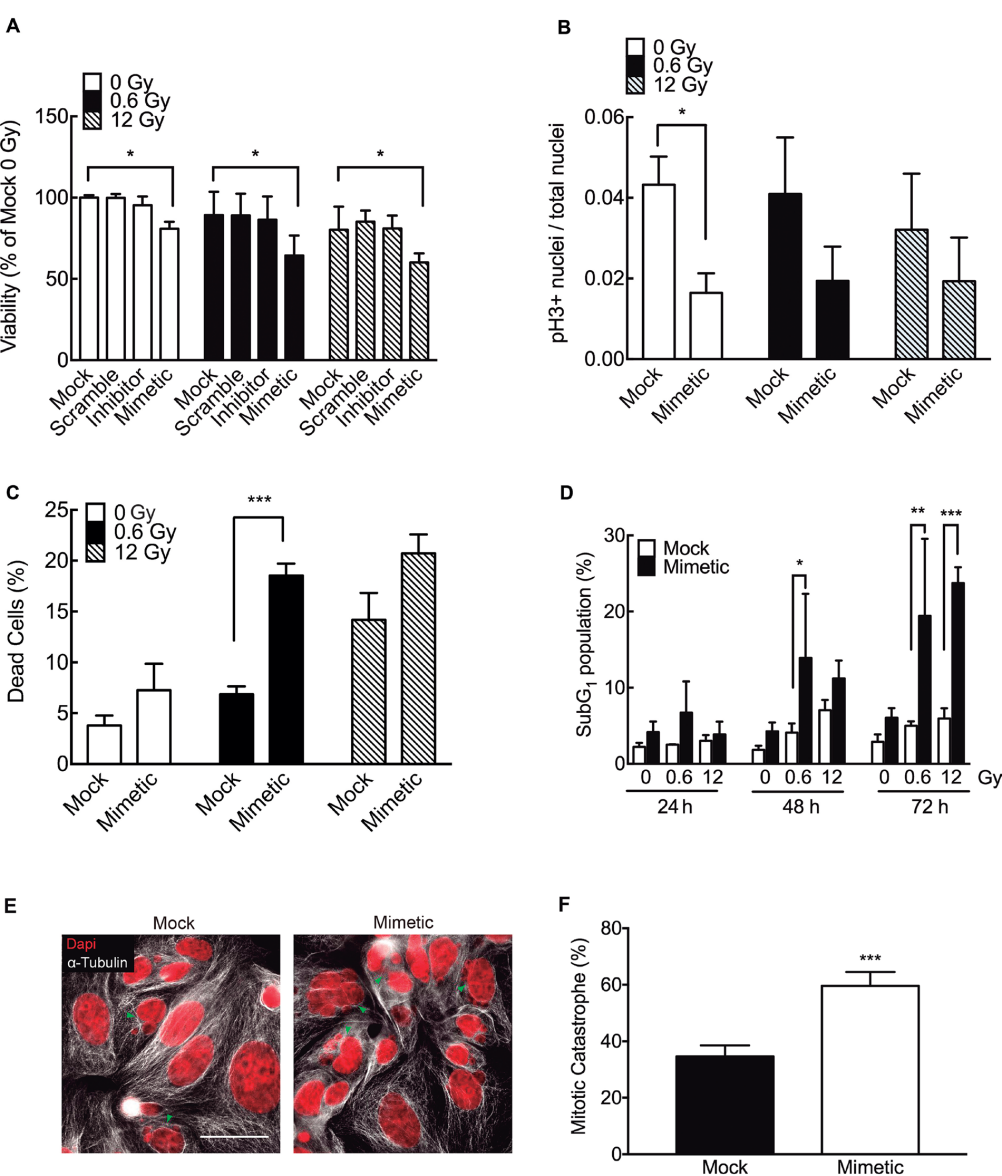
miR-205-3p increases radiosensitivity at low doses of radiation (**A**) DLD-1 cells were mock-transfected or transfected with a scrambled miR control, an anti-miR-205-3p (inhibitor) or a miR-205-3p mimic. Viability was evaluated 48 h post irradiation with 0, 0.6 or 12 Gy, by MTS assay. Results are expressed as a percentage of mock-transfected non-irradiated cells (0 Gy). (**B**) Proliferation (mitotic index) was determined by quantifying cells with positive staining for phosphor-histone H3 in mock and mimetic transfected cells, 48 hours after irradiation. (**C**) Cell death was evaluated by trypan blue exclusion assay in mock and mimetic transfected cells, 48 hours after irradiation. Data represent percentage of cell death in each condition. (**D**) Effect of miR-205-3p on apoptosis was evaluated by quantification of subG1 population in mock and mimetic transfected cells, 24, 48 and 72 hours after irradiation. (**E**) Evaluation of nuclear morphology of mock and mimetic transfected DLD-1 cells, 72 hours after irradiation (12 Gy). Nuclei were stained with DAPI (Red) and cytoplasm with α-tubulin (white). Green head arrows points to fragmented nuclei, features typical of mitotic catastrophe. Scale bar: 50 μm. (**F**) Cells with mitotic catastrophe morphology were counted in 5 different fields and normalized to the number of cells. At least 100 cells were evaluated per sample. Data represent the means ± S.D. of at least 3 independent experiments. ^*^P < 0.05; ^**^P < 0.01; ^***^P < 0.001 using Two way ANOVA (A and D) or *T*-test (B, C and F).

To evaluate whether the most augmented miRNA in response to 0.6 Gy (miR-205-3p) had an effect on radiosensitivity at low doses, DLD-1 cells were transfected with a miR-205-3p mimic. A MTS assay showed that overexpression of miR-205-3p reduced viability in both absence of IR and doses IR of 0.6 and 12 Gy. Transfection of an anti-miR-205-3p (inhibitor) had no effect on viability (Figure 7A). To evaluate the effect of the mimetic miR-205-3p on proliferation, a mitosis specific marker (Phospho-histone H3) was used. Interestingly, overexpression of miR-205-3p significantly reduced proliferation only in the absence of IR (Figure 7B). However, the mimetic miR had no effect on cell cycle distribution either in the control or in the IR cells (Supplementary Figure 1). On the other hand, when the effect of the mimetic miR on cell death was evaluated, a significant but distinct response was observed in IR cells with 0.6 and 12 Gy. At 48 h after IR, cell death increased only in cells exposed to 0.6 Gy (Figure 7C). When the effect of the mimetic miR was evaluated on apoptosis by assessing the sub-G1 population, a significant increase in cell death was observed in cells exposed to 0.6 Gy as early as 24 h after IR (Figure 7D). In addition, the radiosensititizer effect of mimetic miR at 12 Gy was evident only at later time points (72 h) (Figure 7D). Interestingly, with 12 Gy, mimetic miR-205-3p induced formation of multi- and micronucleated cells, features associated with mitotic catastrophe (Figure 7E and 7F). This effect was not seen in cells exposed to 0.6 Gy (data not shown).

**Figure 8:**
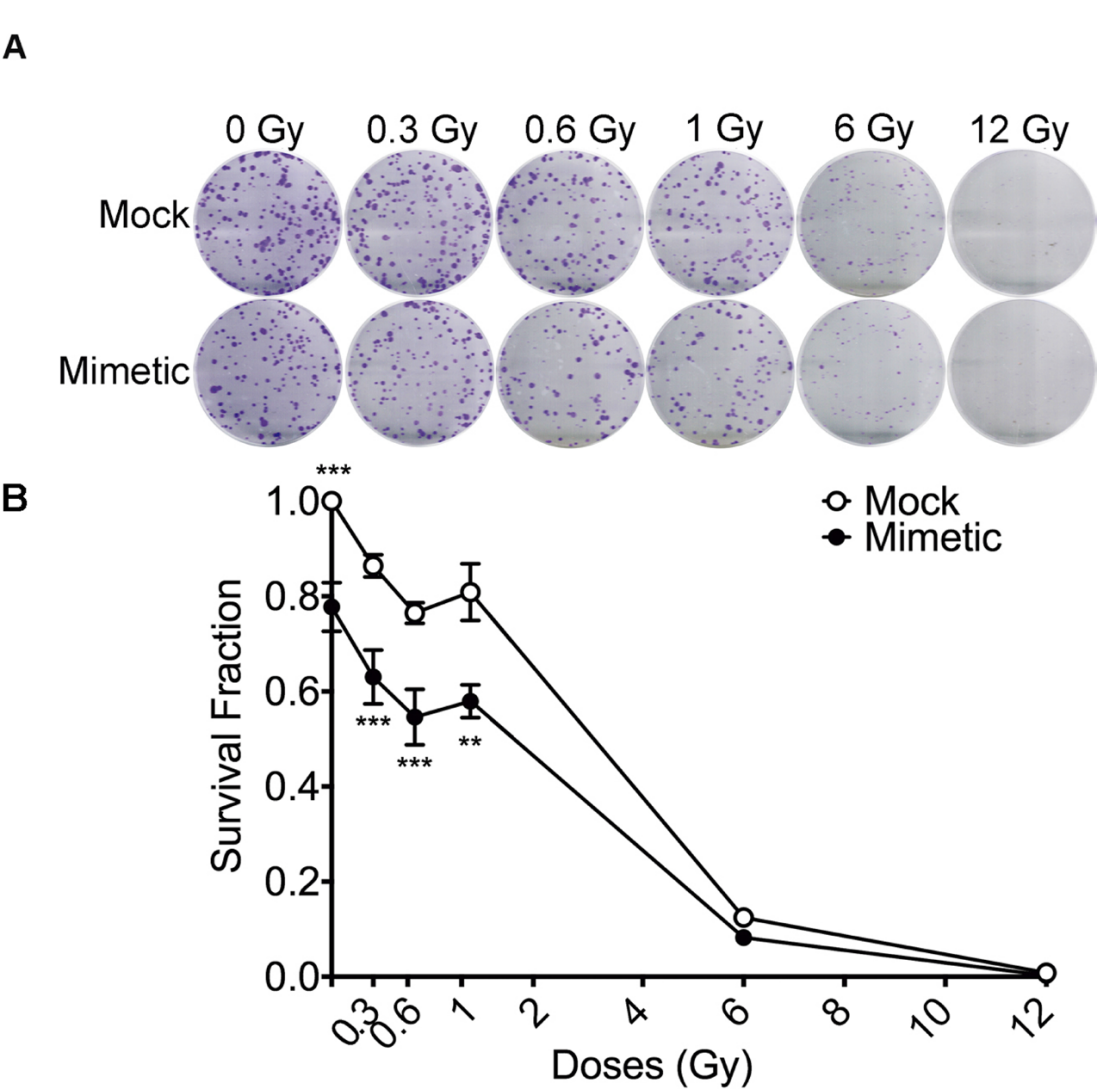
miR-205-3p overexpression increases LDHRS in DLD-1 cells DLD-1 cells were mock-transfected or transfected with a mimetic miR-205-3p 24 hours before irradiation with different doses and clonogenic assay was performed. In (**A**) representative pictures of crystal violet stained colonies obtained under each condition are shown. In (**B**) colonies with diameters ≥200 μm (≈50 cells) were counted using *Gen5* Software and survival fraction was calculated as in Figure 1. Results are expressed relative to the mock-transfected group under non-irradiation conditions (0 Gy). Means ± S.D. of at least 3 independent experiments are shown. ^**^P < 0.01; ^***^P < 0.001, using two-way ANOVA.

In order to confirm the effect of miR-205-3p on LDHRS, DLD-1 cells were transfected with the mimetic miR or control (Mock), and then exposed to different doses of IR and a colony formation assay was performed Consistent with the previous results, miR-205-3p overexpression increased radiosensitivity in about 23% of cells exposed to 0.6 Gy compared to mock-transfected cells (Figure 8). Importantly, transfection with miR-205-3p significantly increased DLD1-1 radiosensitivity only at low-doses range (0–1 Gy), confirming the effect on LDHRS. The radiosensitizer effect of miR-205-3p was also evaluated in other cell lines, with distinct results. In HT29 colon cancer cells, miR-205-3p increased radiosensitivity at doses above 1 Gy, while in MCF7 and MCF10A breast cancer cells, miR-205-3p had no effect on radiosensitivity at any of the analyzed doses (Supplementary Figure 2).

## DISCUSSION

For some time, radiotherapy (RT) has been one of the most often used techniques in cancer treatment. In fact, it is estimated that 60% of patients with solid cancers have received or will receive RT at least once during the course of their illness, and for 15% of these patients, RT is the only treatment that they will receive [1]. In the United States, about 490,000 patients received RT in 2015, and this demand will increase by ∼20% in 2025 [58]. Different devices for radiation delivery are currently used in clinical and RT protocols. These are based on exposing the tumor area to very high doses of radiation in order to induce tumor cell death. However, detrimental effects on surrounding normal tissue and several side effects have been reported [5, 6, 59].

In this context, LDHRS could be a possible way to induce cell death using lower doses than those currently used in RT [25]. In fact, our results show that DLD-1colorectal cancer cells exposed to 0.6 Gy elicit a death response. Even when all the doses that were tested elicited a cell death response (Figure 1B), 1 Gy had a lower effect than 0.6 Gy, which is in accordance with the LDHRS phenomenon [9, 14, 60]. Moreover, survival results at low dose fitted to the induced repair model and not to the LQ one, confirming the LDRHS in these cells [14, 61].

Martin *et al.* noted that ∼75% of the 50 cell lines tested *in vitro* (non-tumor and tumor cells) are LDHRS positive, including malignant cells of colorectal, glioma, breast, prostate, melanoma, bladder, cervix, lung, oral, head and neck cancers. However, LDHRS has not been explored in DLD-1 cells before [9]. To support our analysis, HT-29 cells, previously reported as LDHRS positive at doses of <0.5 Gy [15, 18, 51], were irradiated and 0.3 Gy was found to cause a slight decrease in viability (∼10%, Supplementary Figure 2).

Apoptosis and necrosis are the most common forms of cell death induced by IR, however, the relationship between the dose and a specific type of cell death is still under active investigation [62, 63]. In our model, low doses of 0.6 Gy induced an apoptotic cell death with caspase 3/7 activation. However, although high doses of 12 Gy also induced an activation of these caspases, an important necrotic death component was evidenced 48 h after irradiation. Given that necrotic cell death involves an inflammatory response, frequently reported as an adverse effect of RT [5, 6, 64], the use of radiation protocols that trigger apoptotic cell death (without inflammation) are essential to improve the tumor’s regression.

It has been suggested that one possible mechanism underlying the LDHRS phenomenon is related to the presence of DNA damage [26, 65], specifically double strand breaks (DSB), as described for high doses of ionizing radiation [66, 67]. In our hands, DLD-1 cells exposed to 0.6 Gy showed a significant increase in γ-H2AX foci from 30 min up to 3 h, which also correlated with an increase in some DNA damage response proteins, including pP53, pCHK1 and pCHK2. However, γ-H2AX levels decreased and become similar to control groups 6 h after irradiation (Figure 3A and 3B). On the contrary, γ-H2AX levels were higher and sustained in cells exposed to 12 Gy (Figure 3A and 3B). These results suggest that either DNA damage induced by 0.6 Gy is early repaired or that those cells containing DNA damaged were eliminated. Other studies have shown that both human fibroblast [35] and fibrosarcoma cells [34] display a lineal increase in γ-H2AX foci formation after 0.002 Gy IR. Nevertheless, foci disappear after 8 h in both cell lines. Even in DLD-1 cells exposed to low doses of pulsed X-rays, an early increase in DSB had been reported (30 min), however after 2 h, levels of γ-H2AX foci become similar to unirradiated control [68]. Our findings, according to these reports, support the idea that nuclear DNA damage induced by low IR doses is indeed repaired. A plausible explication for cell death observed even when DNA damage has been repaired; rely on the indirect effect of ionizing radiation on free radical formation and mitochondrial damage. As mitochondrial DNA lacks histones, it is more susceptible to oxidative and radiation-induced damage, that culminate in impaired membrane potential [69, 70]. Indeed, in hippocampal neurons and fibroblast-like cells, low-doses of radiation altered mitochondrial dynamics through upregulation of fission protein dynamin-related protein 1 (Drp1) [71, 72], a protein related with increase of apoptosis [73].

On the other hand, in cells exposed to 12 Gy, γ-H2AX levels and foci were still increased at 48 h after IR, suggesting a distinct IR-induced cell death mechanism for low-dose ranges.

Several studies have evaluated the change in expression of miRNAs at high doses of radiation in colon-derived [39] and other cell lines [48, 74–76], but little information is available about changes in miRNA expression in response to low-dose IR. The evaluation of miRNA expression requires the use of an adequate housekeeping control gene. This implies a reproducible, reliable and stable endogenous control for a correct comparison with the expression of other miRNAs. Most published works evaluating miRNA expression have used small nuclear RNA (i.e. RNU6B, RNU48), however their properties, stability and size are different from miRNAs [77]. Other housekeeping genes frequently used are the miRNAs: let-7, miR-16, miR-423 and miR-374, among others, but the use of a single miRNA to normalize data may induce a systematic error [78]. To minimize data variation, the use of multiple miRNAs has been suggested together with a geometric mean to normalize them [79]. For this reason, our data were normalized by the geometric mean of 6 miRNAs (RNU6B, RNU5G, miR-423-5p, miR191-5p, miR-16-5p and let-7a). These miRNAs were analyzed using NormFinder [80] and they showed the lowest variation between samples (data not shown).

In order to validate PCR-array results, we evaluated the expression of some target genes belonging to the most differentially expressed miRNAs (VEGF-A, PKCε, MMP9 and NOD2 as targets of miR-205-3p, miR-1, miR-133b and miR-122-5p, respectively). These targeted mRNAs were chosen for their role in oncogenesis and because they have been previously validated in the literature, with the exception of VEGF-A (a miR-205-3p target). As we expected, targets of the overexpressed miRNAs displayed a significant down-regulation (Figure 6A and 6C). Although levels of expression of PKCε did not reach a significant difference, a slight decrease was evident when compared to the control and 12 Gy. Nevertheless, PKCε was validated as a target of miR-1 in cardiac ischemia (in mouse model) [55], therefore, it is plausible that miR-1 could be targeting different mRNAs in colorectal cancer.

The most increased miRNA at low doses, miR-205-3p, is expressed together with miR-205-5p mostly in breast, prostate and thymus cancers [81] as well as both miRNAs are significantly increased in non-small cell lung carcinoma and squamous cell carcinoma [82]. Most publications have focused on miR-205-5p and its dual role in cancer. MiR-205-5p has been reported as oncomiR in lung [83] and nasopharyngeal cancers [84] by targeting PTEN [85]. Also a role as a tumor suppressor has been described for miR-205-5p in prostate [86, 87], breast [88], melanoma [89], glioblastoma [90] and colon cancers [91] by targeting cMYC [92], PKCε [86], and VEGF-A [90]. Interestingly, in DLD-1 cells exposed to low doses of radiation (<1 Gy) miR-205-3p reduced proliferation and induced cell death with an important apoptotic component. Even when little information is available for miR-205-3p in the literature, with no experimental validated targets, *in silico* analysis for predicting targets of miR-205-3p showed that 17 out of 33 validated targets for miR-205-5p could also be targets for miR-205-3p (DDX5, ZEB1, BCL2, VEGF-A, ESRRG, KCNJ10, SMAD4, ERBB3, AR, LRRK2, YES1, SMAD1, ACSL4, PTEN, HMGB3, PHLPP2, YY1) suggesting a similar function for both miRNAs (miRbase and TargetScan 7.1). Additionally, DDX3X gen (DEAD-box polypeptide 3) was the target with higher probability to be regulated by miR-205-3p. In breast cancer, DDX3X had been associated with ephitelial-mesenchymal transition [93], while in gallbladder cancer promote metastasis to lymphatics nodes [94]. In HCT116 and HT-29 colon cancer cells, inhibition of DDX3X reduces proliferation, presumably by involving the Wnt pathway [95–97].

MiR-122-5p was the most decreased miRNA in response to 0.6 Gy. In healthy livers, miR-122 is elevated and represents 70% of the total miRNA pool while it is diminished in liver tumors [98]. Over-expression of miR-122 in liver cells suppresses proliferation and induces apoptosis [99–101]. The functions of miR-122 in colon cancer cells or its role in radiosensitivity have not been documented. However, an increase in miR-122 expression was reported in liver metastasis from colorectal cancer although was undetectable in primary tumors or in normal mucosa from patients [102]. This result suggests that miR-122 may act as an oncomiR in colorectal cancer and, therefore, may be down-regulated at low doses of radiation in DLD-1 cells.

MiR-134-5p was another miRNA whose levels displayed a major decrease in response to 0.6 Gy. MiR-134-5p is a brain-enriched miRNA, with an important role in the development and differentiation of the vertebrate central nervous system [103]. Overexpression of miR-134 promotes proliferation and inhibition of apoptosis in lung cancer [104] as well as head and neck squamous cell carcinoma cells [105], while in glioblastoma [106] and colorectal cancer cells [107] has the opposite effect, promoting apoptosis and reducing proliferation. In addition, differential miRNA expression has also been reported in human B lymphoblastic [108] and peripheral blood mononuclear cells [47] exposed to different IR doses. This suggests that levels of miRNAs are IR dose-dependent since they are a reflection of differential gene expression and pathway activation. The exact function of miR-134 in response to IR is unknown and further studies are necessary to determine its role as an oncomiR or tumor suppressor.

MiR-218-5p, a tumor suppressor miRNA, was the most over-expressed miRNA in response to 12 Gy. This miRNA is down-regulated in different cancers [109] and transfection with a miR-218-5p mimic inhibits migration and proliferation in non-small-cell lung cancer cells by targeting the epidermal growth factor receptor (EGFR) [110]. In colorectal cancer cells, miR-218-5p reduced migration, invasion and colony formation by targeting the Metastasis Associated with Colon Cancer 1 protein (MACC1) [111].

Interestingly, miR-1 was the only miRNA that significantly increased at both 0.6 and 12 Gy IR doses. MiR-1 is abundantly expressed in cardiac and skeletal muscle tissue [112] and its expression is down-regulated in several solid cancers: testes, colon, lung, ovary, lymphoma and prostate [113].

In HT-29 and HTC-116 colorectal cancer cells, miR-1 acts as a tumor suppressor, reducing proliferation and migration by targeting the c-*MET* oncogene (Hepatocyte growth factor receptor) [114], a member of the MAPK pathway [115]. Similarly, Xu *et al.* showed that overexpression of miR-1 in HT-29 and Caco2 cells suppressed aerobic glycolysis by targeting Smad3, a critical protein for HIF-1α signaling, causing a reduction in proliferation [116]. Furthermore, transfection with miR-1 in cardiomyocytes induces apoptosis by targeting the anti-apoptotic protein Bcl-2 [117]. All this data suggests that, in DLD-1 cells, miR-1 may be inducing apoptosis in response to low and high-dose radiation.

Interestingly, in cells exposed to 12 Gy, miR-205-3p displayed an augmented number of multi- and micronuclei, a morphological feature associated with mitotic catastrophe [118, 119]. This observation suggests that at low doses miR-205-3p induce cell death by apoptosis, but at high doses could augment mitotic catastrophe. Here, the effect of miR-205-3p was also evaluated in different cell lines (Supplementary Figure 2), finding distinct effects, at different doses, in each cell line analyzed. Although confusing, this data reinforce the concept that radiation sensitivity is not driven solely by gene expression, but rather by a combination of distinct parameters, including cell type and radiation dose [120].

To date, most studies have evaluated the effect of different miRNAs on radiosensitivity using high doses of radiation (> 1 Gy), however this strategy does not consider the adverse effect observed in clinical protocols. Neglia *et al.* reported an increased relative risk of 0.69 per Gy for all central nervous system tumors when a cohort of 5-year survivors of childhood cancer were exposed at doses >1 Gy/daily [121]. *In vitro* experiments showed a lineal relationship between transformation and radiation doses ranging from ∼1 to ∼4–5 Gy, while no transformation effect was detected below 0.1 Gy [122]. In this context, LDHRS appears as an opportunity to improve the therapeutic effect of RT while reducing the adverse effects. We found that miR-205-3p, the most augmented miRNA in response to 0.6 Gy, could significantly increase the radiosensitivity of DLD-1 cells, maximizing the LDHRS phenomenon.

The precise mechanism that miR-205-3p uses to trigger apoptosis is unknown, nor are its targets in colon cancer cell lines. Further functional analysis will be necessary to elucidate the role of differentially expressed miRNAs in LDHRS, as well as the molecular mechanism involved in its expression under these ionizing radiation conditions. Meanwhile, the data presented here might contribute to strengthen efforts on building algorithms based on the integration of gene expression data and other biological and clinical parameters, aimed to predict the radiosensitivity and enhancing the outcome for each particular patient [36, 120, 123–127].

## MATERIALS AND METHODS

### Cell culture and viability assays

DLD-1 human colorectal cancer cells were cultured in RPMI1640 (Mediatech, Herndon, VA, USA) supplemented with 10% heat-inactivated fetal bovine serum (Mediatech), penicillin G (100 U/ml), and streptomycin (100 µg/ml) and incubated in a 5% CO_2_ humidified atmosphere at 37° C. Media was changed every 3 days and cells were trypsinized when they reached 80%–90% confluence. Cells were seeded in 96-well plates at a density of 2 × 10^3^ cells/well. After ionizing radiation, 20 µL of MTS plus 1 µL PMS (Cell Titer 96 Aqueous, Promega, Madison, Wl, USA) were added to each well, followed by incubation for 3 hours at 37° C. The plates were mixed for 30 seconds and absorbance was measured at 490 nm (Cytation 3™, BioTek instruments, Winooski, VT, USA). Viability was also evaluated by mixing cells with an equal volume of 0.4% trypan blue solution (Logos Biosystems, Gyunggi-Do, Korea). Cells were counted using a LUNA™ Automated Cell Counter (Logos Biosystem). All assays were performed at least three times.

### Cell irradiation

Cells were irradiated with ^60^Co sources at the Comisión Chilena de Energía Nuclear (CCHEN). For doses lower than 1 Gy, a Noratom 3500 at 0.15 Gy/min was used. For high doses, a Gammacell 220R (Nordion) at 20 Gy/min was used. After irradiation, cells were split for the following assays.

### Caspase 3/7 and citotoxicity assay

Citotoxicity and caspase activity was measured using the ApoTox-Glo Triplex assay kit (Promega, Madison, WI, USA). Briefly, 100 µL of viability/citotoxicity buffer was added to 2 × 10^3^ cells on 96-well plates. The mixture was incubated for 30 minutes at room temperature, and fluorescence was determined using a microplate reader (Ex 485 nm/Em 520 nm) (Cytation 3™, BioTek instruments). After reading, 100 µL of caspase 3/7 substrate (Z-DEVD) was added and the mixture was incubated for 30 minutes at room temperature, and luminescence was evaluated.

### Indirect immunofluorescence

Two-days after irradiation, cells grown on glass coverslips were fixed using 4% p-formaldehyde for 10 minutes, permeabilized with 0.25% Triton X-100 at room temperature and blocked with 3% BSA/PBS for 45 minutes. Cells were incubated overnight with an anti-γ-H2AX antibody (1:1000, Millipore, Temecula, CA, 05-636), anti-phospho Histone H3 (Ser10, 1:1000, Millipore, Temecula, CA, 06-570) or anti-α-tubulin (1:5000, ThermoFisher Scientific, Illinois, USA, PA5-22060) prepared in 0.05% Triton X-100 and 1% BSA/PBS. After washing, cells were incubated with an anti-mouse Alexa Fluor-488 secondary antibody (1:500, Molecular Probes, A-21042) or anti-rabbit Alexa Fluor-488 secondary antibody (1:500, Invitrogen A-11034). Slides were mounted with ProLong Gold Antifade Reagent with DAPI (Life Technologies, NY). Cells were photographed under a fluorescence microscope (BX53; Olympus, Japan). For γ-H2AX quantification, foci were counted using a Find Maxima plugin and normalized by nuclei numbers using ImageJ software (Rasband, National Institutes of Health, USA). Every focus bigger than 0.1 μm (13 pixels) was considered as positive.

### Cell cycle analysis

A day before irradiation, 2.4 × 10^4^ cells/well on 6-well plates were seeded. After irradiation, cells were collected by trypzinization at 24, 48 and 72 h, and then fixed with 70% ethanol at 4° C for 24 h. Cells were stained with a DNA staining solution (50 µg/mL of Propidium Iodide, Sodium citrate 1% (w/v), 1% NP-40, 10 µg/mL of Rnase A and PBS 1X). Cell cycle distribution was evaluated by fluorescence-activated cell sorter FACSCanto (BD) and data was analyzed using FlowJo software (Treestar, Inc., San Carlos, CA).

### Real-time quantitative PCR

Total RNA was extracted using Trizol reagent (Ambion, Austin, TX). MicroRNA cDNAs were prepared using mIRCURY LNA Universal RT microRNA (Exiqon, Denmark). Expression of a subset of 86 miRNAs was evaluated in an array platform (Supplementary Table 1) using ExiLENT SYBR Green master mix (Exiqon). Data were normalized by the geometric mean of: RNU6B, RNU5G, miR-423-5p, miR191-5p, miR-16-5p and let-7a. For miRNA profiling, a 2-fold increase or decrease with adjusted *P* < 0.05 was considered significant.

For validation studies, cDNAs were prepared using Affinity Script qPCR (Agilent Technologies, Santa Clara, CA, USA) and quantified using Brillant II Sybr (Agilent) in an Eco^™^ Real-time PCR System (Illumina Inc., San Diego, CA, USA). The target’s mRNA levels were normalized to HPRT levels and expressed as 2^–ΔΔCt^. Data represent at least three independent experiments and each sample was measured in duplicate. The specific primers used for qPCR are show in Supplementary Table 2.

### Western blotting

Cells were lysed in a RIPA buffer and 30 g proteins were loaded and separated in sodium dodecyl sulfate–polyacrylamide electrophoresis (SDS–PAGE) gels and transferred onto nitrocellulose membranes (Thermo Scientific, Rockford, IL, USA). After blocking with TBS-T containing 5% BSA, membranes were incubated overnight at 4° C with the following antibodies: Anti γ-H2AX (1:1000, Millipore, Temecula, CA), phospho-P53 (1:2000), P53 (1:2000), phospho-Chk1 (1:2000), phospho-Chk2 (1:2000), Chk1 (1:2000) and Chk2 (1:2000) from Cell Signaling (Danvers, MA). Anti-β-actin (1:10.000, Sigma, St Louis, MO) was used as a loading contrtol.

### Transient transfection of cells with miR-205-3p mimic

Cells were seeded in 6-well plates (6 × 10^4^ cells per well) in 500 µL of RPMI-1640 plus supplements and transfected with 5 nM of a miR-205-3p mimic (purchased from Qiagen, Crawley, UK) using HiPerFect Transfection Reagent (Qiagen) according to the manufacturer’s protocol. miR-205-3p transfection efficiency was assessed by qPCR from 24 h to 72 h post-transfection and by transfecting a EGFP-tagged small RNA.

### Prediction of mRNAs targeted by miR-205-3p

In order to predict the potential miR-205-3p´s targets, algorithms from miRbase (http://www.mirbase.org) and TargetScan 7.1 (http://www.targetscan.org/vert_71) were used.

### Clonogenic survival assay

DLD-1, HT29, MCF7 and MCF10a cells transfected with the mimetic miR-205-3p were seeded at a density of 500-1000 cells per 6-wells plate and 3 hours later, exposed to 0, 0.3, 0.6, 1, 6 and 12 Gy. Irradiated cells were incubated for 10–14 days and then fixed with 10% of p-formaldehyde for 10 minutes and stained with 0.5% crystal violet for 30 minutes. Excess of dye was raised with PBS and the number of colonies, greater than 50 cells, was counted as surviving colonies, using the *Gen5* software (supplied with the Biotek Cytation3). The survival fraction at each doses were calculated as previously described [10, 128].

### Data analysis for survival assays

The surviving fraction (*S*) data at all doses tested, were fitted to linear-quadratic (LQ) and induced-repair (IR) model (Equation 1), using nonlinear least squares regression through the iterative method of Gauss-Newton with step halving (Python Software).

$$S\left(D\right)=\exp\left\{-\alpha D-\beta D^{2}\right\}\left[61,129\right]$$ Equation 1

S(D)=exp{−ar[1+(asar−1)e−DDc]D−βD2}[11,21−23]; Equation 2

where *d* is the dose of radiation used, *a*_*r*_, is the slope extrapolated from high dose response, *a*_*S*_ is the survival curve slope measured at low doses, *Dc* is the transition from LDHRS to IRR response and β is a constant, as in the linear-quadratic equation

### Statistical analysis

Data are represented as the means ± S.D. of at least three independent experiments. The statistical significance between experimental and control groups was calculated using two-way ANOVA followed by Dunnet’s multiple comparision test. For miRNA profiling, the statistical significance between irradiated and control groups was analyzed using Student’s *t* test and Adjusted *P*-value was calculated using Sidak-Bonferroni post-test. Graphpad Prism V6.0 (GraphPad Software, San Diego, CA) was used to perform all analyses and graph data. A *P* < 0.05 was considered significant.

## SUPPLEMENTARY MATERIALS FIGURES AND TABLES


