# Supplementary material for: Differential miRNA expression profiling reveals miR-205-3p to be a potential radiosensitizer for low- dose ionizing radiation in DLD-1 cells
Source: Oncotarget. 2018 May 29;9(41):26387–405. doi: 10.18632/oncotarget.25405 (PMC5995186; doi:10.18632/oncotarget.25405)
Supplement: Supplementary file 1 [file oncotarget-09-26387-s001.pdf]

Differential miRNA expression profiling reveals miR-205-3p to be a potential radiosensitizer for low- dose ionizing radiation in DLD-1 cells

SUPPLEMENTARY MATERIALS

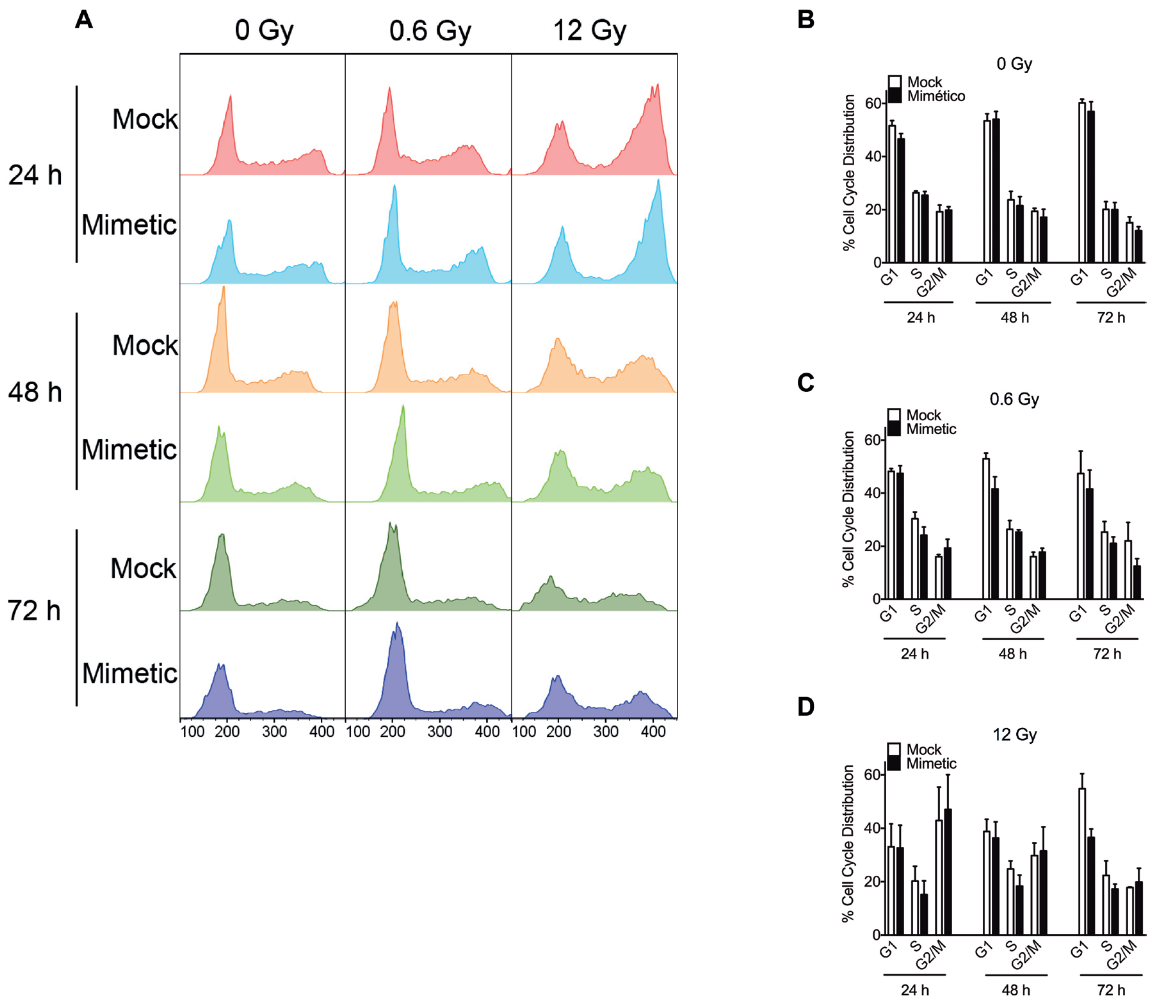

Supplementary Figure 1: Cell cycle distribution of DLD-1 cells at different times post irradiation in cells transfected with a miR-205-3p mimic or mock-transfected. Results are expressed as a percentage of cell cycle distribution at each time.

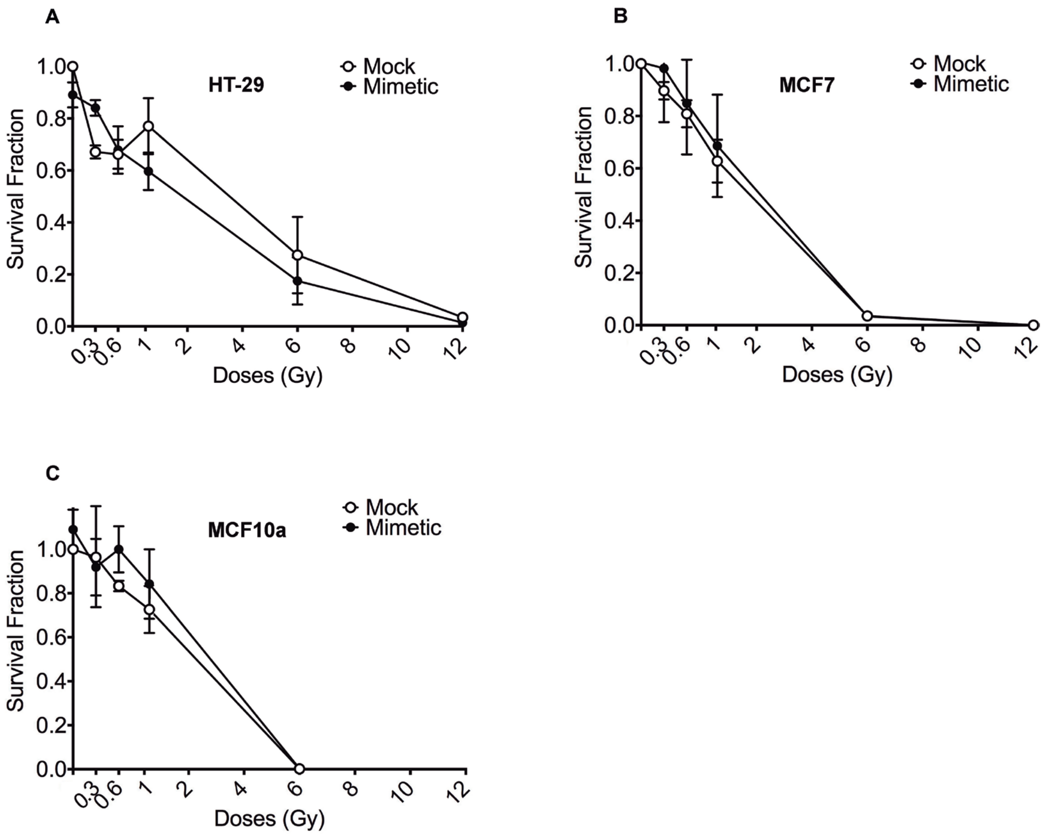

Supplementary Figure 2: Effect of miR-205-3p on survival of (**A**) HT29, (**B**) MCF7 and (**C**) MCF10a cells, evaluated by clonogenic assay at 10–14 days post irradiation. Colonies ≥200 µm (≈50 cells) were counted using *Gen5* Software. Survival fraction was calculated as described in methods.

Supplementary Table 1: List of miRNAs evaluated by array-PCR

miRNA name	Sequence	miRNA name	Sequence
hsa-miR-106a-5p	AAAAGUGCUUACAGUGCAGGUAG	hsa-miR-218-5p	UUGUGCUUGAUCUAACCAUGU
hsa-miR-141-3p	UAACACUGUCUGGUAAAGAUGG	hsa-miR-26a-5p	UUCAAGUAAUCCAGGAUAGGCU
hsa-miR-183-5p	UAUGGCACUGGUAGAAUUCACU	hsa-miR-26b-5p	UUCAAGUAAUUCAGGAUAGGU
hsa-miR-210-3p	CUGUGCGUGUGACAGCGGCUGA	hsa-miR-27a-3p	UUCACAGUGGCUAAGUUCCGC
hsa-miR-214-3p	ACAGCAGGCACAGACAGGCAGU	hsa-miR-30c-5p	UGUAAACAUCCUACACUCUCAGC
hsa-miR-338-3p	UCCAGCAUCAGUGAUUUUGUUG	hsa-miR-30e-5p	UGUAAACAUCCUUGACUGGAAG
hsa-miR-378a-3p	ACUGGACUUGGAGUCAGAAGGC	hsa-miR-31-5p	AGGCAAGAUGCUGGCAUAGCU
hsa-miR-98-5p	UGAGGUAGUAAGUUGUAUUGUU	hsa-miR-365a-3p	UAAUGCCCCUAAAAAUCCUUAU
hsa-miR-133a-3p	UUUGGUCCCCUUCAACCAGCUG	hsa-miR-409-3p	GAAUGUUGCUCGGUGAACCCCU
hsa-miR-181a-5p	AACAUUCAACGCUGUCGGUGAGU	hsa-miR-449a	UGGCAGUGUAUUGUUAGCUGGU
hsa-miR-21-5p	UAGCUUAUCAGACUGAUGUUGA	hsa-miR-708-5p	AAGGAGCUUACAAUCUAGCUGGG
hsa-miR-25-3p	CAUUGCACUUGUCUCGGUCUGA	hsa-miR-101-3p	UACAGUACUGUGAUAACUGAA
hsa-miR-30a-5p	UGUAAACAUCCUCGACUGGAAG	hsa-miR-29b-3p	UAGCACCAUUUGAAAUCAGUGUU
hsa-miR-30b-5p	UGUAAACAUCCUACACUCAGCU	hsa-miR-149-3p	AGGGAGGGACGGGGGCUGUGC
hsa-miR-125a-5p	UCCCUGAGACCCUUUAACCUGUGA	hsa-miR-153-3p	UUGCAUAGUCACAAAAGUGAUC
hsa-miR-125b-5p	UCCCUGAGACCCUAACUUGUGA	hsa-miR-15b-5p	UAGCAGCACAUCAUGGUUUACA
hsa-miR-20a-5p	UAAAGUGCUUAUAGUGCAGGUAG	hsa-miR-16-5p	UAGCAGCACGUAAAUAUUGGCG
hsa-miR-622	ACAGUCUGCUGAGGUUGGAGC	hsa-miR-181b-5p	AACAUUCAUUGCUGUCGGUGGGU
hsa-miR-30d-5p	UGUAAACAUCCCCGACUGGAAG	hsa-miR-195-5p	UAGCAGCACAGAAAUAUUGGC
hsa-let-7c-5p	UGAGGUAGUAGGUUGUAUGGUU	hsa-miR-203a	GUGAAAUGUUUAGGACCACUAG
hsa-let-7g-5p	UGAGGUAGUAGUUUGUACAGUU	hsa-miR-204-5p	UUCCCUUUGUCAUCCUAUGCCU
hsa-miR-1	UGGAAUGUAAAGAAGUAUGUAU	hsa-miR-29a-3p	UAGCACCAUCUGAAAUCGGUUA
hsa-miR-101-3p	UACAGUACUGUGAUAACUGAA	hsa-miR-29c-3p	UAGCACCAUUUGAAAUCGGUUA
hsa-miR-122-5p	UGGAGUGUGACAAUGGUGUUUG	hsa-miR-34a-5p	UGGCAGUGUCUUAGCUGGUUGU
hsa-miR-133b	UUUGGUCCCCUUCAACCAGCUA	hsa-miR-497-5p	CAGCAGCACACUGUGGUUUGU
hsa-miR-193b-5p	CGGGGUUUUGAGGGCGAGAUGA	hsa-miR-512-5p	CACUCAGCCUUGAGGGCACUUUC
hsa-miR-194-5p	UGUAACAGCAACUCCAUGUGGA	hsa-miR-134-5p	UGUGACUGGUUGACCAGAGGGG
hsa-miR-204-5p	UUCCCUUUGUCAUCCUAUGCCU	hsa-miR-221-3p	AGCUACAUUGUCUGCUGGGUUUC
hsa-miR-491-5p	AGUGGGGAACCCUUCCAUGAGG	hsa-miR-423-5p	UGAGGGGCAGAGAGCGAGACUUU
hsa-miR-512-5p	CACUCAGCCUUGAGGGCACUUUC	hsa-miR-32-5p	UAUUGCACAUUACUAAGUUGCA
hsa-miR-542-3p	UGUGACAGAUUGAUAACUGAAA	hsa-miR-148b-5p	AAGUUCUGUUAUACACUCAGGC
hsa-miR-9-5p	UCUUUGGUUAUCUAGCUGUAUGA	hsa-miR-148b-5p	AAGUUCUGUUAUACACUCAGGC
hsa-miR-146a-5p	UGAGAACUGAAUUCCAUGGGUU	hsa-miR-124-3p	UAAGGCACGCGGUGAAUGCC
hsa-miR-181c-5p	AACAUUCAACCUGUCGGUGAGU	hsa-miR-18a-5p	UAAGGUGCAUCUAGUGCAGAUAG
hsa-miR-181d-5p	AACAUUCAUUGUUGUCGGUGGGU	hsa-miR-196b-5p	UAGGUAGUUUCCUGUUGUUGGG
hsa-miR-92a-3p	UAUUGCACUUGUCCCGGCCUGU	hsa-let-7a-5p	UGAGGUAGUAGGUUGUAUAGUU
hsa-miR-128-3p	UCACAGUGAACCGGUCUCUUU	hsa-miR-145-5p	GUCCAGUUUUCCCAGGAAUCCCU
hsa-miR-143-3p	UGAGAUGAAGCACUGUAGCUC	hsa-miR-15a-5p	UAGCAGCACAUAAUGGUUUGUG
hsa-miR-144-3p	UACAGUAUAGAUGAUGUACU	hsa-miR-17-5p	CAAAGUGCUUACAGUGCAGGUAG
hsa-miR-200c-3p	UAAUACUGCCGGGUAAUGAUGGA	hsa-miR-185-5p	UGGAGAGAAAGGCAGUUCCUGA
hsa-miR-205-5p	UCCUUCAUUCCACCGGAGUCUG	hsa-miR-192-5p	CUGACCUAUGAAUUGACAGCC
hsa-miR-206	UGGAAUGUAAGGAAGUGUGUGG	hsa-miR-451a	AAACCGUUACCAUUACUGAGUU
hsa-miR-212-3p	UAACAGUCUCCAGUCACGGCC	hsa-miR-33a-5p	GUGCAUUGUAGUUGCAUUGCA
hsa-miR-423-5p	UGAGGGGCAGAGAGCGAGACUUU	hsa-miR-191-5p	CAACGGAAUCCCAAAAGCAGCUG
hsa-miR-520d-5p	CUACAAAGGGAAGCCCUUUC		

Supplementary Table 2: List of primers used in RT-qPCR

Target	Forward primer	Reverse primer
VEGF-A	ATGCGGATCAAACCTCACCA	ACACGTCTGCGGATCTTGTA
PKCε	ACTGGCAAGTTTGCTGGGAA	CAACGAACACGCAAGCCTTT
MMP9	AGAGCTTGTCCCGGTCGTAGT	GGCAATGCTGATGGGAAACCCT
NOD2	TTGTGAGCAGGCTGTGAGTT	TGGCAAAAGTAGGCTTGGGA
HPRT	TGCTTTCCTTGGTCAGGCAGTA	CAACACTTCGTGGGGTCCTTTT
